# Cloning and Functional characterization of the promoter of a high affinity potassium transporter gene from *Eucalyptus grandis*

**DOI:** 10.1186/1753-6561-5-S7-P163

**Published:** 2011-09-13

**Authors:** Carolina Costa, Flávio Tetsuo Sassaki, Juliana Bravo, Ivan Maia

**Affiliations:** 1 IB - UNESP – Botucatu, Brazil; 2 IB-UNESP-Botucatu, Brazil

## 

The characterization of organ/tissue-specific promoters is of great interest to transgenic production. The construction of expression cassettes containing tissue-specific promoters is a viable alternative to limited transgene expression to specific organs and cell types. In this context, the purpose of this study was to functionally characterize the promoter of a *Eucalyptus grandis* gene encoding a high affinity potassium transporter (named EgHAK) shown to be specifically expressed in roots. For that, the 5’-flanking region of *EgHAK* (1,3 kb) was cloned and transcriptionally fused to the b-glucuronidase reporter gene (GUS), and then used to transform tobacco leaf discs. Histochemical analysis of GUS activity in transgenic plants showed that GUS staining was mainly detected in vascular tissues of leaf and root. To investigate the response of the studied promoter to potassium starvation, a hydroponic system was employed. In this case, enhanced GUS staining was observed in the roots of plants starved for 6 days when compared to control ones. Moreover, a weak induction of the promoter at low potassium conditions was observed using fluorimetric assays. Thus, our results indicate that, in a heterologous system, the studied promoter shows preferential expression in roots in the absence of potassium.

